# Multifunctional Cross-Linked Shrimp Waste-Derived Chitosan/MgAl-LDH Composite for Removal of As(V) from Wastewater and Antibacterial Activity

**DOI:** 10.1021/acsomega.2c07391

**Published:** 2023-03-08

**Authors:** Rachid El Kaim Billah, Zineb Azoubi, Eduardo Alberto López-Maldonado, Hicham Majdoubi, Hassane Lgaz, Eder C. Lima, Anita Shekhawat, Youssef Tamraoui, Mahfoud Agunaou, Abdessadik Soufiane, Ravin Jugade

**Affiliations:** † Department of Chemistry, Faculty of Sciences, Laboratory of Coordination and Analytical Chemistry, University of Chouaib Doukkali, El Jadida 24000, Morocco; ‡ Laboratory of Physiopathology and Molecular Genetics, Faculty of Sciences Ben M’Sick, Hassan II University of Casablanca, Casablanca 20450, Morocco; § Faculty of Chemical Sciences and Engineering, Autonomous University of Baja, California, CP, Tijuana 22390, Baja California, Mexico; ∥ Materials Science energy and Nanoengineering Department (MSN), Mohammed VI Polytechnic University (UM6P), Lot 660-Hay Moulay Rachid, Benguerir 43150, Morocco; ⊥ Innovative Durable Building and Infrastructure Research Center, Center for Creative Convergence Education, Hanyang University-ERICA, 55 Hanyangdaehak-ro, Sangrok-gu, Ansan-si, Gyeonggi-do 15588, Republic of Korea; # Institute of Chemistry, Federal University of Rio Grande do Sul, Porto Alegre 91501-970, RS, Brazil; ∇ Department of Chemistry, RTM Nagpur University, Nagpur 440033, India

## Abstract

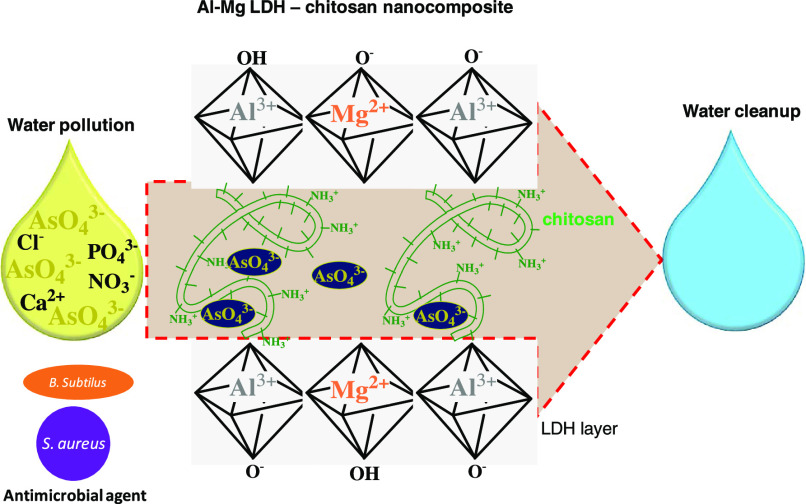

This work synthesized a novel chitosan-loaded MgAl-LDH (LDH = layered double hyroxide) nanocomposite, which was physicochemically characterized, and its performance in As(V) removal and antimicrobial activity was evaluated. Chitosan-loaded MgAl-LDH nanocomposite (CsC@MgAl-LDH) was prepared using cross-linked natural chitosan from shrimp waste and modified by Mg–Al. The main mechanisms predominating the separation of As(V) were elucidated. The characteristic changes confirming MgAl-LDH modification with chitosan were analyzed through Fourier transform infrared spectroscopy, X-ray diffraction, thermogravimetric analysis-differential thermal analysis, and Brunauer-Emmett-Teller measurements. Porosity and the increased surface area play an important role in arsenic adsorption and microbial activity. Adsorption kinetics follows the general order statistically confirmed by Bayesian Information Criterion differences. To understand the adsorption process, Langmuir, Freundlich, and Liu isotherms were studied at three different temperatures. It was found that Liu’s isotherm model was the best-fitted model. CsC@MgAl-LDH showed the maximum adsorption capacity of 69.29 mg g^–1^ toward arsenic at 60 °C. It was observed that the adsorption capacity of the material rose with the increase in temperature. The spontaneous behavior and endothermic nature of adsorption was confirmed by the thermodynamic parameters study. Minimal change in percentage removal was observed with coexisting ions. The regeneration of material and adsorption–desorption cycles revealed that the adsorbent is economically efficient. The nanocomposite was very effective against *Staphylococcus aureus* and *Bacillus subtilus*.

## 1 Introduction

The physicochemical properties of arsenic play an important role in various industries, but it is a toxic heavy metal that seems difficult to not use.^1^ Since it is non-biodegradable, continuous utilization increases its accumulation in the environment. Moreover, arsenic can cause complications in various organ systems like the nervous, respiratory, endocrine, and renal systems if a human being is exposed to it.^2,3^ So there is a need to develop an efficient remediation methodology for the removal of As from the environment.^3−6^ Among various techniques, adsorption is a prime attraction because of its cost-effectiveness and better efficiency for removing most of the toxicants.^7^ There are lots of advanced functional materials used for the adsorption process.^8−10^ Biodegradability and ample availability of various biomaterials attract researchers to utilize them as adsorbents. Sometimes these biosorbents are used directly or in a modified form. Cross-linking,^11,12^ impregnation,^13^ and composite^14^ formation of biopolymers lead to the fabrication of novel adsorbents with enhanced physicochemical properties. Biopolymers, like cellulose,^15^ chitin,^16^ alginate,^17^ chitosan,^18^ fluorapatite natural,^19^ etc., were studied in the present scenario. Among them, chitosan shows wide applications, as it has amino and hydroxyl group functionalities to combine with organic and inorganic moieties.^6,20−22^ Layered double hydroxides (LDHs) have stacked layers with a positive charge and can act as an anion exchangers in the interlayer region.^4,8,9,23−25^ High surface area and an ionic surface compared to other nanosize materials lead to thermodynamically unstable agglomeration of LDH.^26^ This property of LDHs acts as a stumbling block for some specific applications. To overcome this, surface modification,^27^ defects introduction,^28^ and building hybrid materials^29^ were common strategies reported in the literature.^8^ On the other hand, nanocomposites formed by incorporating layered inorganic materials like LDHs with polymers show remarkable changes in chemical and physical properties,^30,31^ including high surface area, thermal and mechanical stability, and flexibility.^9,32^ In the literature the use of MgAl-LDH for the removal of perchlorooctanoic acid,^33^ chromate,^34^ and phosphate ions^35^ proves that MgAl-LDH can be used as potent adsorbent for anionic species in native or hybrid form. MgAl-LDH shows selective adsorption toward arsenate ion depending on the molar ratio of aluminum and magnesium, nitrate ion orientation, and experimental conditions. The adsorption capacity of MgAl-LDH toward arsenic varied in the range of 5–615 mg/g as reported in previous studies. Therefore, MgAl-LDH can be used as a potent and selective adsorbent for scavenging arsenic in industrial wastewater and drinking water.^36^ Since chitosan has very low adsorption capacity toward arsenic,^37^ its capacity can be increased by combination with LDHs. The modified chitosan-based materials with LDH have more selectivity toward the pollutants.^38^ A composite of MgAl-LDH and chitosan has not been reported for selective arsenic removal from water. So, in the present work, a nanocomposite has been fabricated by incorporating MgAl-LDHs in glutaraldehyde cross-linked chitosan having an affinity toward arsenic, and its antimicrobial activity was explored. In addition, parameters important for adsorption were optimized. With these optimized conditions, the adsorption of arsenic has been studied. As a result, the material shows good adsorption capacity toward arsenic. Moreover, easy handling and regeneration of material make the material cost-effective.

## 2 Methods and Materials

### 2.1 Chemicals

The chemicals and reagents used for the synthesis and adsorption process were of analytical reagent (AR) grade and used further without any purification. Magnesium nitrate, aluminum nitrate, sodium hydroxide, glutaraldehyde, nitric acid, hydrochloric acid, sodium chloride, sodium arsenate dibasic heptahydrate (Na_2_HAsO_4_·7H_2_O), sulfuric acid, acetic acid, and ethanol were procured from Sigma-Aldrich.

### 2.2 Preparation of CsC@MgAl-LDH

1 g of chitin from shrimp shells was refluxed and stirred for 6 h with 48% (w/V) sodium hydroxide to obtain the chitosan (CsC). The product obtained was filtered, washed, and dried.^39,40^

MgAl-LDH (1:2) was prepared by the coprecipitation method; for this, 100 mL of an aqueous solution of Mg(NO_3_)_2_·6H_2_O, Al (NO_3_)_3_·9H_2_O, and NaNO_3_ was prepared, and sodium hydroxide was added to the mixture for adjusting the pH to 10. The precipitate obtained along with the supernatant was kept for 24 h for maturation. A centrifugation process was used to collect the precipitate. The obtained precipitate of MgAl-LDH was washed with distilled water and methanol. Finally, it was oven-dried at 70 °C for 12–14 h.

**Figure 1:**
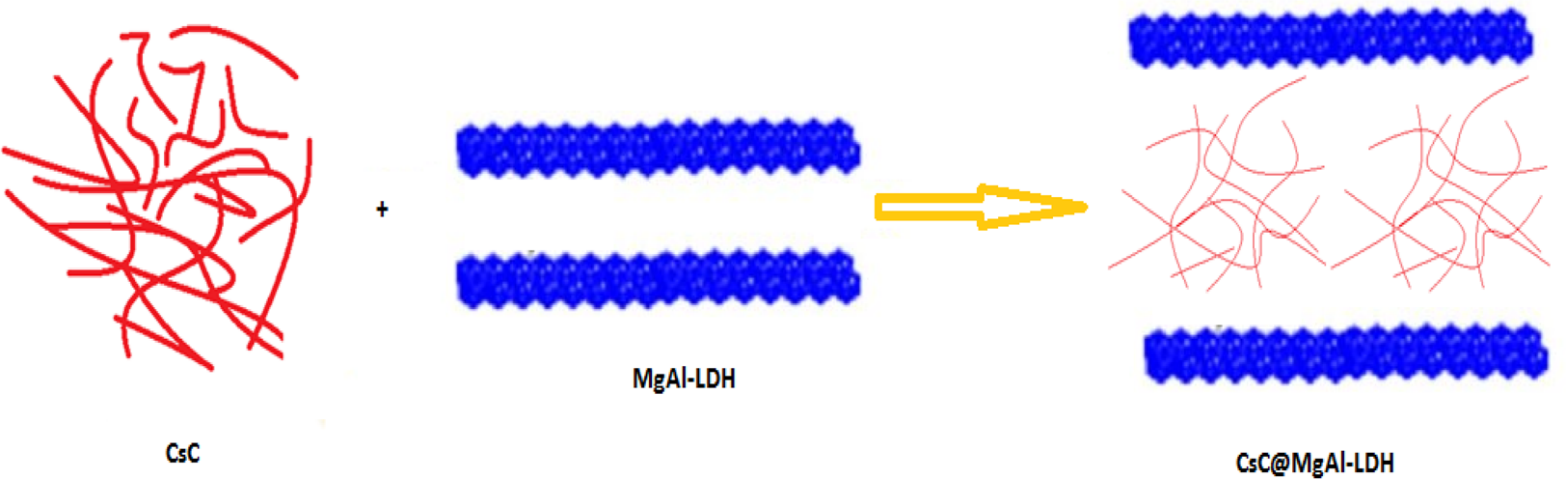
Schematic presentation–Synthesis of CsC@MgAl-LDH composite.

In a 100 mL solution of 3 g of CsC in 1% v/v of acetic acid, 10 mL of 0.025 M glutaraldehyde and 1 g of MgAl–LDH powder were added simultaneously.The mixture was stirred for 6 h and treated with sodium hydroxide for precipitation. The resulting precipitate was washed thoroughly with distilled water and then oven-dried. Figure 1 shows the schematic presentation of CsC@MgAl-LDH.

### 2.3 Physicochemical Characterization

X-ray diffraction (XRD), Bruker D8 Advance, Billerica, MA, USA, was used for the recording of XRD patterns. A Philips XL 30 ESEM (Acc spot Magn 20.00 kV) was utilized for observing changes in the surface specifics of the adsorbent as compared to the native material. New active functional groups that were introduced were identified by Fourier transform infrared (FT-IR) spectroscopy, PerkinElmer 2000, Waltham, MA, USA. A thermogram of the material was obtained by thermogravimetric analysis (TGA)/differential thermogravimetry (DTG), STD Q 600, Artisan Technology Group, Kansas City, MO, USA, operated with heating rate of 10 °C/min under a nitrogen atmosphere.

### 2.4 Adsorption Studies

Adsorption of As(V) on CsC@MgAl-LDH was studied in a batch adsorption process. For this, 100 mL of arsenic solutions was equilibrated with 0.1 g of adsorbent in an Erlenmeyer flask at pH 7.0. The flasks were capped and stirred for 5–180 min in a thermostatic shaker at 25°, 40°, and 60 °C. The following mathematical equations were used to calculate the amount of As(V) uptake *q*_e_ (mg/g) at equilibrium and the percentage of As(V) removal.
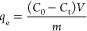
1
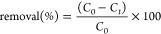
2Here, *C*_o_ and *C*_t_ are the concentration of initial and retained As(V) in the solution at time *t* (mg/L), respectively, *V* is the solution volume (L), and *m* is the mass of adsorbent (g).^5^

### 2.5 Antibacterial Assay

The antibacterial activity of CS/R Mg–Al was tested through a well-diffusing technique. First, Mueller–Hinton (MH) agar plate surfaces were inoculated with the tested strains previously adjusted to 0.5 McFarland turbidity standards. Next, a well with a diameter of 6 mm was punched aseptically with a sterile tip, introducing a volume of 100 μL of the antimicrobial agent. Then, the plates were incubated for 24 h at 37 °C.^20,41−43^ The antimicrobial activity was detected by measuring the zone of inhibition after the incubation.

The test bacteria are the Gram-negative bacteria *Escherischia coli* ATCC8739 and *Pseudomonas aeruginosa* ATCC27853, and the Gram-positive bacteria *Staphylococcus aureus* ATCC6538 and *Bacillus subtilus* ATCC6633.

## 3 Results and Discussion

### 3.1 Physicochemical Characterization

**Figure 2:**
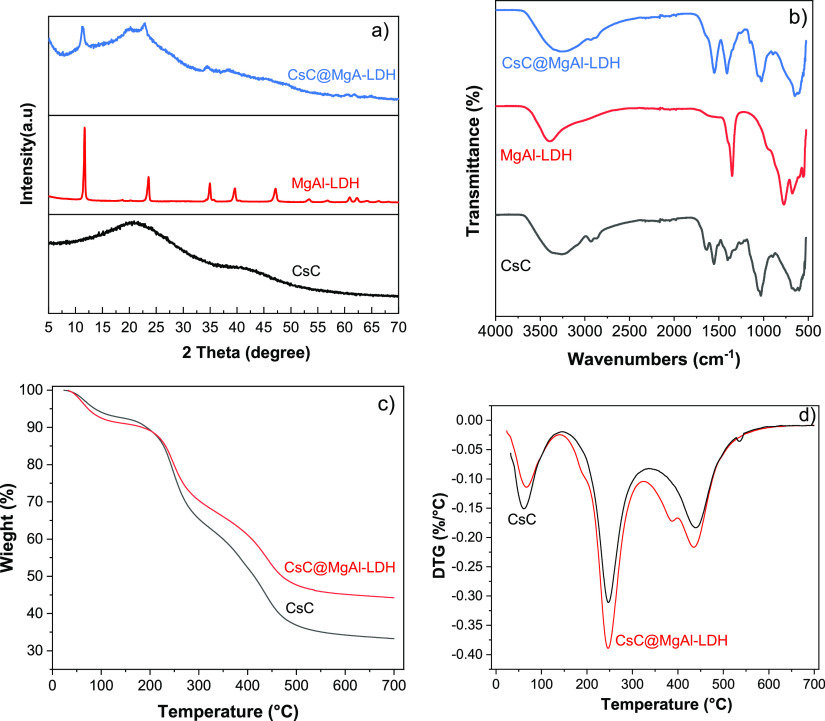
(a) XRD pattern and (b) FT-IR spectra of CsC, MgAl-LDH, and CsC@MgAl-LDH. (c) TGA and (d) DTA curves of CsC and CsC@MgAl-LDH.

In XRD spectra (Figure 2a), it was observed that the characteristic peaks of chitosan disappeared in glutaraldehyde cross-linked chitosan, showing a broad peak centered at 2θ = 20°.^44^ In MgAl-LDH, at lower 2θ values, strong diffraction peaks are attributed to characteristic reflections of crystalline nature.^21,45^ LDH-loaded cross-linked chitosan shows peaks of the 003 and 012 planes of LDH and a broad peak of cross-linked chitosan, confirming the formation of LDH-loaded cross-linked chitosan.

In FT-IR spectra (Figure 2b) of cross-linked chitosan, a peak at 1559 cm^–1^ for the amide II band was observed, indicating a glutaraldehyde cross-linked chitosan.^39,44^ The NH and OH stretching vibrations give a broad peak at 3417 cm^–1^.^39^ Symmetrical stretching of CH_3_ and C=O stretching were observed at 2937 and 1645 cm^–1^, respectively. In the case of Mg–Al LDH, the peak at 3451 cm^–1^ was due to OH vibrations.^8,46,47^ A peak at 1389 cm^–1^ is attributed to the NO_3_ vibrations. Al–O and Mg–O vibrations show peaks at 713 and 548 cm^–1^, respectively.^46,48,49^ In the case of CsC@MgAl-LDH, characteristic peaks of cross-linked chitosan along with a peak at 1389 cm^–1^ of MgAL-LDH LDH and peaks due to M-O linkages in the range of 600–750 cm^–1^ have been observed, which confirms the formation of the material.^6,9^

Scanning electron microscopy (SEM) micrographs of CsC (Figure S1a) show a rough and porous morphology due to chitosan cross-linking with glutaraldehyde. Granules observed with a rough surface in CsC@MgAl-LDH confirm the incorporation of LDH in the CsC, increasing the surface’s scaffolds (Figure S1b). In CsC@MgAl-LDH (Figure S1b), the respective peaks of elements for CsC and MgAl-LDH were clearly observed in EDS spectra.

The surface area of CsC@MgAl-LDH was 318.26 m^2^ g^–1^, more than that of CsC, which is 96.09 m^2^ g^–1^. Decreases were noticed in the average pore diameter from 7.86 to 5.50 nm after the modification of cross-linked chitosan, which confirms the incorporation of LDH into the polymer matrix (see Figure S2b,a).^8,9,45^ Since the pores’ size was between 2 and 50 nm, it confirms the mesoporous behavior of the material.^6,21,39^ The hysteresis loop shown in Figure S2a was of H4 type, which proves the complexity of the material.^50^ The Barrett-Joyner-Halenda (BJH) pore volume curve is shown in Figure S2b. The increased surface area contributes to the higher adsorption capacity of the material.^21,49^

TGA-DTA of CsC and CsC@MgAl-LDH (Figure 2c) were studied in the temperature range of 25–700 °C. The TGA thermogram of CsC@MgAl-LDH showed an overall mass loss of 50%. The mass loss of 10% in 30–125 °C represents evaporation of moisture content. After that, until 200 °C, the composite remains stable. Between 200 and 500 °C, 40% loss in weight was observed due to the destruction of cross-linked chitosan.^21,45,46^ Above 500 °C, the biopolymer was completely destroyed leaving behind stable oxides of magnesium and aluminum oxides.^51^ In the DTA analysis of CSC, a first endothermic peak was observed in the range of 25–150 °C due to moisture removal (Figure 2d). The endothermic peak between 150 and 350 °C is attributed to breaking cross-linked chitosan chains with glutaraldehyde.^9,39^ The third peak in the range of 370–500 °C may be due to degradation of chitosan polymeric chains into the oligomeric form.^44^ In the case of CsC@Mg–Al LDH composite, it was observed that there was shifting in all three peaks. These variations in the peak positions were due to different interactions of cross-linked chitosan with MgAl-LDH.^25,44,46^ These minor changes revealed the suitable biocompatibility of cross-linked chitosan with LDH.^5^

### 3.2 Adsorption Studies

#### 3.2.1 Effect of pH

**Figure 3:**
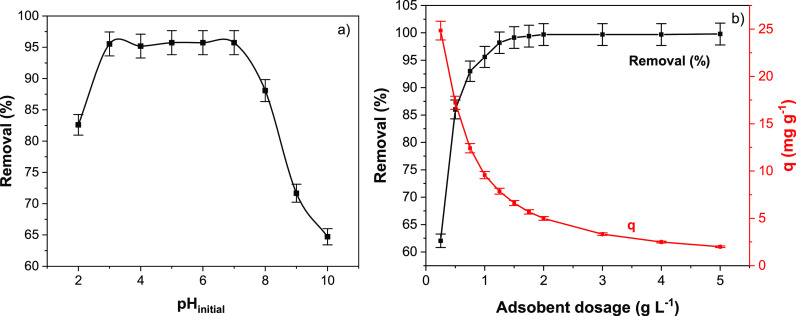
Effect of (a) pH and (b) adsorbent dose on removal (%) of As(V).

The initial pH of the adsorbate solution could be mandatory for conducting the adsorption experiments. Using the CsC@MgAl-LDH adsorbent, the uptake of arsenic was practically constant from pH 3 to 7 (Figure 3a). At pH 4, 97.97% of As(V) is present in H_2_AsO_4_^–^.^3,49^ Therefore, this species should be the most adsorbable among all the possible As(V) species (see section 3.7 and Figure 9).

#### 3.2.2 Effect of the Mass of the Adsorbent

The effect of the adsorbent dosage is presented in Figure 3b. This figure presents two important points to be discussed. First, as the adsorbent dosage increases, the percentual removal of As(V) increases continuously until the adsorbent dosage of 1.5 g L^–1^ (99.15% removal) is kept constant until the adsorbent dosage of 5 g L^–1^. Conversely, the sorption capacity decreased continuously as the adsorbent dosage was increased. In this sense, it is necessary to establish an adsorbent dosage that does not impair the sorption capacity (*q*). The decrease of *q* with the increase of adsorbent dosage occurs by two main factors.

- First, the unsaturation of adsorption sites due to an increase in adsorbent mass at fixed amount of adsorbate and volume in the adsorption process.^5^
- Second, there was decrease in the adsorbent surface area and increase in diffusional path length due to larger mass of adsorbent. This leads to reduction in adsorbent capacity because of particle aggregation.^5^

Besides these two points, mathematically, the sorption capacity (*q*) is inversely proportional to the adsorbent dosage (X), as depicted in the equation below.
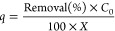
3Based on these features, an adsorbent dosage of 1 g L^–1^, which attained a maximum removal of 95.60% and did not decrease the sorption capacity remarkably, was chosen to continue this research.^5^

### 3.3 Kinetics of Adsorption

**Figure 4:**
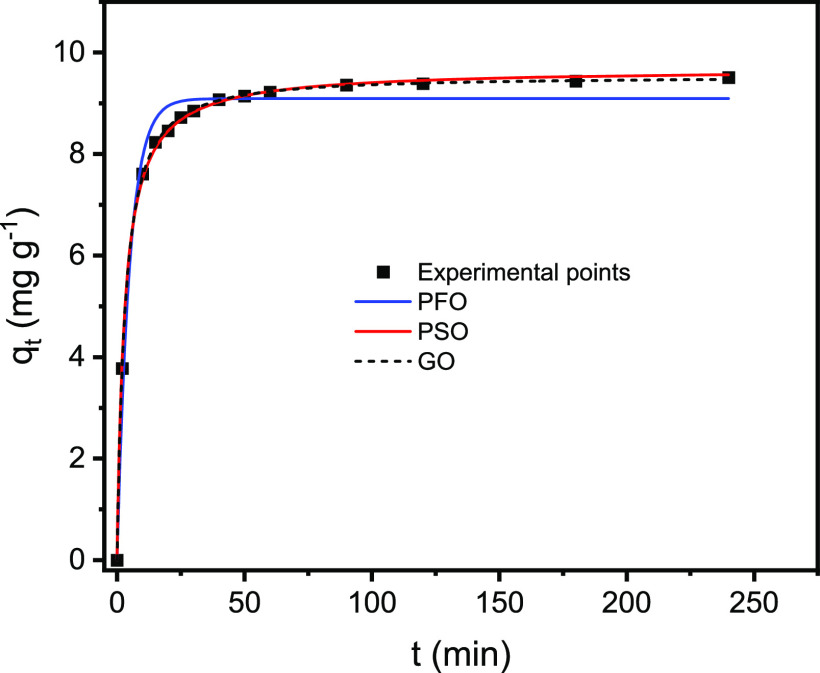
Nonlinear fitting of PFO, PSO, and GO for the uptake of 10 mg L^–1^ As(V).

The nonlinear pseudo-first-order (PFO), pseudo-second-order (PSO), and general-order (GO) kinetic adsorption models^52^ were studied to understand the kinetics of As(V) adsorption onto CsC@MgAl-LDH. The results were depicted in Table 1 and Figure 4.

**Table 1:** Kinetic Parameters for Adsorption of As(V) onto the CsC@MgAl-LDH Adsorbent^a^

Kinetic parameters	
Pseudo-first-order	
*q*_e_ (mg g^–1^)	9.093
*k*_1_ (min^–1^)	0.2062
*t*_1/2_ (min)	3.361
*t*_0.95_ (min)	14.53
*R*^2^ adjusted	0.9811
SD (mg g^–1^)	0.3724
BIC	–21.90
Pseudo-second-order	
*q*_e_ (mg g^–1^)	9.681
*k*_2_ (g mg^–1^ min^–1^)	3.560.10^–2^
*t*_1/2_ (min)	2.833
*t*_0.95_ (min)	44.40
*R*^2^ adjusted	0.9991
SD (mg g^–1^)	0.079 63
BIC	–65.09
General-order	
*q*_e_ (mg g^–1^)	9.523
*k*_N_ (min^–1^.(g mg^–1^)^*n*–1^)	0.052 65
*n*	1.793
*t*_1/2_ (min)	2.909
*t*_0.95_ (min)	35.80
*R*^2^ adjusted	0.9998
SD (mg g^–1^)	0.036 05
BIC	–85.86

a Conditions: the initial adsorbate concentration was 10.6 mg L^–1^, the temperature was fixed at 25°C, the adsorbent dosage of 1.0 g L^–1^, initial pH of the adsorbate solution was 4.0.

The kinetic models were statistically evaluated using *R*^2^_adj_, the standard deviation of the residues (SD), and the Bayesian Information Criterion (BIC). The general-order kinetic model found to be most fitted, as values of *R*^2^_adj_ were closer to 1. The lowest values of SD indicate that the sorption capacity values at time *q*_t_ obtained experimentally are closer to the *q*_t_ fitted by the model. The ΔBIC between GO and PFO was 63.96, whereas the ΔBIC between GO and PSO was 20.77. Therefore, undoubtedly, the kinetics of the adsorption process can be well-explained by a GO kinetic model.

The kinetic models are useful for defining the time for attaining equilibrium in an adsorption process. Nevertheless, different kinetic models present different constant rates that have different units. In this sense, it is useful to employ *t*_1/2_ and *t*_0.95_ as kinetic parameters. These terms mean the time necessary to attain 50% and 95% of the saturation. Their values are obtained by interpolation in the fitted curve, using the highest value of *q*_t_ as 100% of saturation (*q*_100_). Using values of 50%*q*_100_ and 95%*q*_100_ as the *Y*-value, it is interpolated in the fitted curve, and we obtained the values of *t*_1/2_ and *t*_0.95_. Considering that the best kinetic model was the GO to describe the adsorption kinetics of As(V), it can be assumed that 2.909 and 35.80 min are the *t*_1/2_ and *t*_0.95_, respectively.

The minimum time to attain the equilibrium in the isotherm studies should surpass *t*_0.95_; therefore, the equilibrium isotherm had 50 min as the contact time between the adsorbent and the adsorbate to perform the isotherm studies.

### 3.4 Equilibrium and Thermodynamics of Adsorption

**Figure 5:**
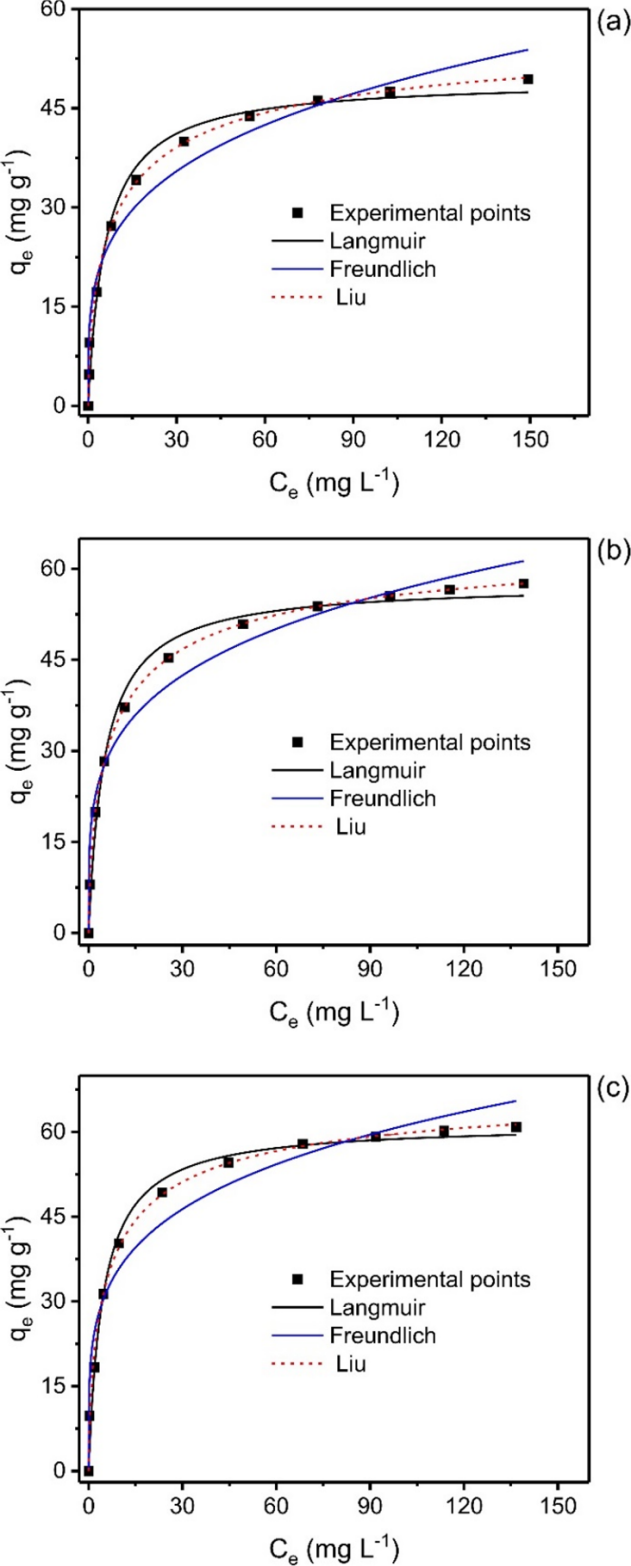
Nonlinear fitting of isotherms. (a) 25, (b) 40, and (c) 60 °C. Conditions: the adsorbent dosage of 1.00 g L^–1^, pH 4.00, and contact time of 50 min.

Langmuir, Freundlich, and Liu isotherm models were studied for As(V) adsorption onto CsC@MgAl-LDH at three different temperature (25, 40, and 60 °C) (Table 2 and Figure 5). These isotherm models^39,53^ were statistically evaluated using *R*^2^_adj_, SD, and BIC values. According to Table 2, the Liu isotherm model presented the *R*^2^_adj_ closer to 1, the lowest values of SD and BIC. Besides that, the ΔBIC values between Liu and Langmuir and Liu and Freundlich ranged from 13.80 to 88.62 and 24.39–97.90, respectively. These values of ΔBIC ≫ 10 between Liu and Langmuir and Liu and Freundlich indicate that certainly, the Liu isotherm model was the best-fitted equilibrium model.

**Table 2:** Langmuir, Freundlich, and Liu Isotherm Parameters for the Adsorption of As(V) on CsC@MgAl-LDH^a^

**As(V)**			
	**25 °C**	**40 °C**	**60 °C**
**Langmuir**			
*Q*_max_ (mg g^–1^)	49.29	57.61	61.49
*K*_L_ (L mg^–1^)	0.1684	0.1949	0.2199
*R*^2^_adj_	0.9809	0.9871	0.9871
SD (mg g^–1^)	2.536	2.344	2.508
BIC	25.46	23.73	25.21
**Freundlich**			
*K*_F_ (mg·g^–1^·(mg·L^–1^)^–1/nF^)	14.64	18.79	21.35
*n*_F_	3.845	4.175	4.388
*R*^2^_adj_	0.9642	0.9703	0.9622
SD (mg g^–1^)	3.470	3.558	4.299
BIC	32.36	32.91	37.07
**Liu**			
*Q*_max_ (mg g^–1^)	58.79	66.74	69.29
*K*_g_ (L mg^–1^)	0.099 49	0.1248	0.1589
*n*_L_	0.6263	0.6434	0.6643
*R*^2^_adj_	0.9965	0.9999	0.9967
SD (mg g^–1^)	1.089	0.039 71	1.274
BIC	7.967	–64.88	11.41

a The adsorbent dosage of 1.0 g L^–1^, initial pH of the adsorbate solution was 4.0.

**Figure 6:**
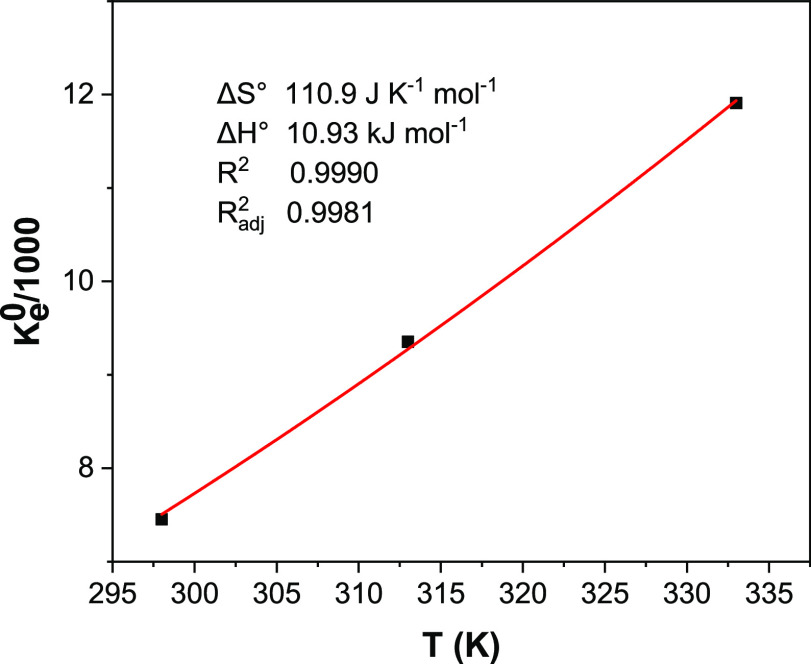
Nonlinear van’t Hoff plot for determination of the thermodynamic parameters of adsorption.

From the best-fitted isotherm model (Liu), the thermodynamic equilibrium constant^54^ was obtained. The nonlinear van’t Hoff equation^55^ was used for calculating thermodynamic parameters (Table 3 and Figure 6).

**Table 3:** Thermodynamic Parameters of the Adsorption of As(V) on CsC@MgAl-LDH

**Temperature (K)**	**298**	**313**	**333**
**Liu model**			
*K*_e_^0^	7.454 × 10^3^	9.353 × 10^3^	1.191 × 10^4^
Δ*G*° (kJ mol^–1^)	–22.09	–23.79	–25.98
Δ*H*° (kJ mol^–1^)	10.93		
Δ*S*° (J K^–1^ mol^–1^)	110.9		
*R*^2^	0.9990		
*R*^2^_adj_	0.9981		

The thermodynamic adsorption data confirmed that the adsorption process was spontaneous, as Δ*G*° values were less than 0, endothermic (Δ*H*° > 0), and favorable (elevated values of *K*_e_^0^). The values of Δ*S*° were positive, indicating that As(V) species adsorbed were more organized in the adsorbed phase than in the aqueous solution.

### 3.5 Effect of Co-ions

**Figure 7:**
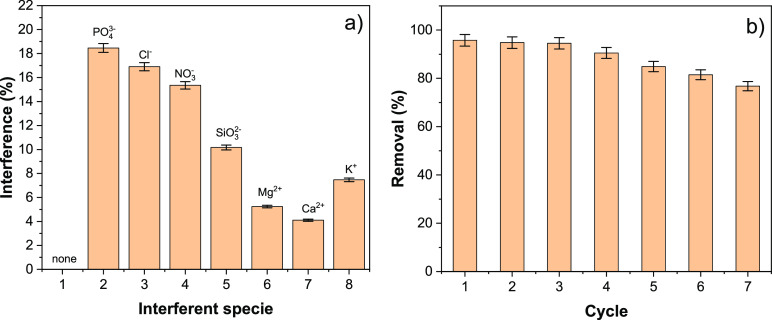
(a) Effects of competitive anions and (b) adsorption–desorption cycles.

Cl^–^, SiO_3_^2–^, NO_3_^–^, PO_4_^3–^, Ca^2+^, K^+^, and Mg^2+^ with varied concentration range (0.1–1.0 mmol/L) were used to understand the effect of coexisting ions during adsorption of arsenic. Figure 7a shows that NO_3_^–^, PO_4_^3–^, and Cl^–^ decrease the total percentage removal by 15–18%. However, in the case of SiO_3_^2–^, As(V) removal was decreased by 10%. Cations show a reduction in removal percentage by 4–8%. Anions tend to reduce the adsorption capacity of the material to some extent.

### 3.6 Regeneration of Adsorbent

0.01 mol/L sodium hydroxide solution was used for regeneration of the spent CsC@MgAl–LDH. Figure 7b shows that material can be reused to adsorb As(V) up to four regeneration cycles. There was minimal change in the percentage removal of As(V) on CsC@MgAl–LDH from the first to fourth adsorption–desorption cycles, that is, from 87 to 85%. The absorbent was stable during the regeneration, confirmed by the aluminum and magnesium leaching test, in the treated medium used for adsorption.^5,60,61^ In Table 4, the adsorption capacity is compared with respect to other reported adsorbent materials, showing the high performance of this new biomaterial.

**Table 4:** Comparison of As(V) Uptake Capacity

**Adsorbent**	***q***_**m**_, mg/g	**Ref**
Chitosan red Scoria	0.72	(56)
Chitosan pumice blends	0.71	(56)
carboxymethylchitosan@ Fe_3_O_4_	20.0	(57)
chitosan coated bentonite	67.11	(58)
chitosan-coated kaolinite	64.89	(58)
chitosan-coated sand	16.78	(58)
chitosan titanium adsorbent	14.4	(59)
CsC@MgAl-LDH	69.29	This work

### 3.7 Physicochemical Performance and Elucidation of As (V) Removal Mechanisms with the Mg–Al LDH-Chitosan Nanocomposite

**Figure 8:**
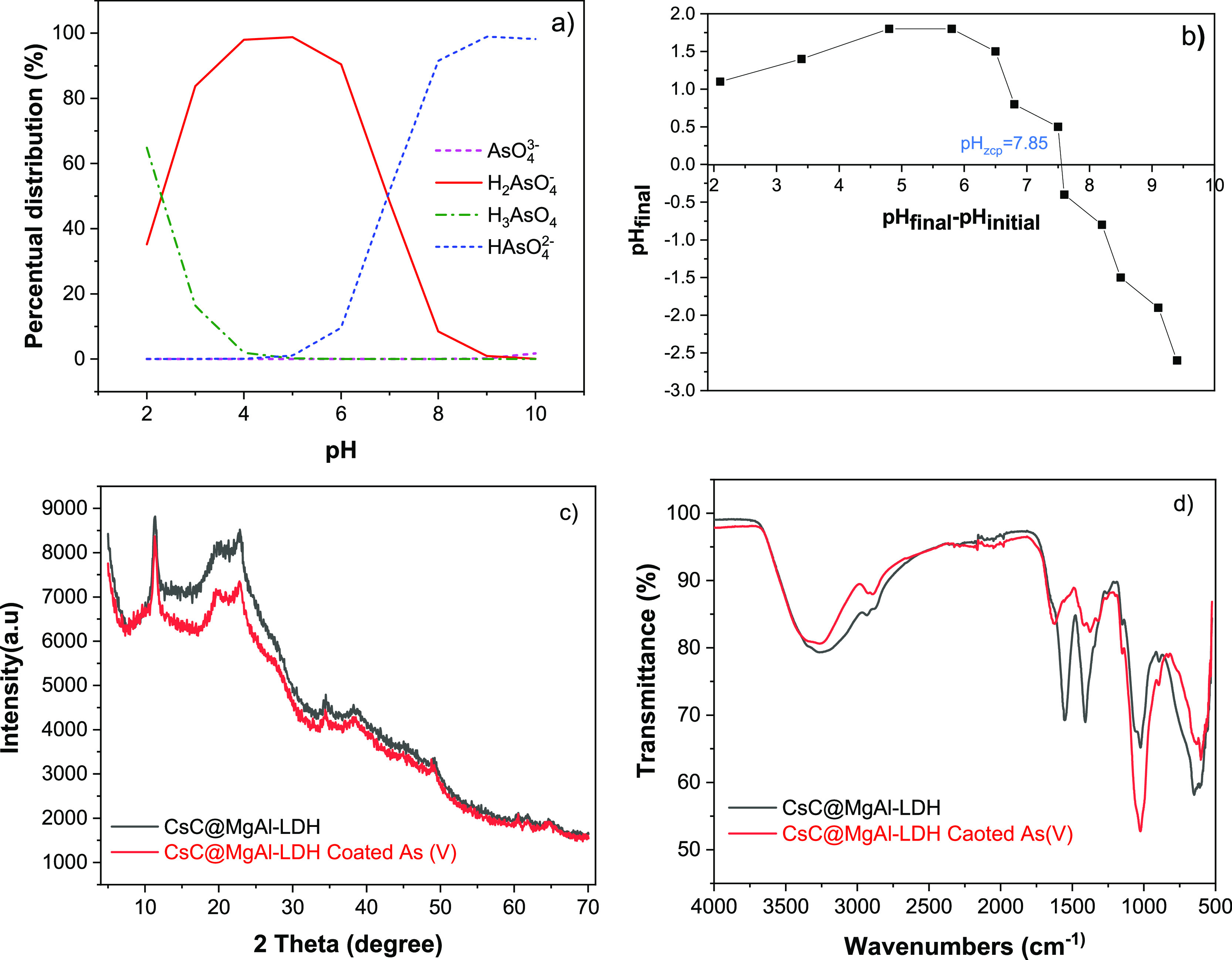
(a) Diagram of zones of the predominance of arsenic species as a function of pH. (b) Zero charge point of the adsorbent. (c) XRD patterns and (d) FTIR before and after adsorption of As(V) on MgAl-LDH-Chitosan.

The pH is a key parameter to understand the physicochemical behavior of the MgAl-LDH-chitosan nanocomposite against As(V) anions. The chemical speciation of As is a function of its main p*K*_a_ values present in an aqueous medium.^3,61,62^ The distribution (%) of the predominant species as a function of pH is observed in Figure 8a. At pH values < p*K*_a1_ (2.3), the fully protonated As species predominates, as do the surface functional groups of the LDH layers and the polyelectrolyte chains of chitosan.^9^

**Figure 9:**
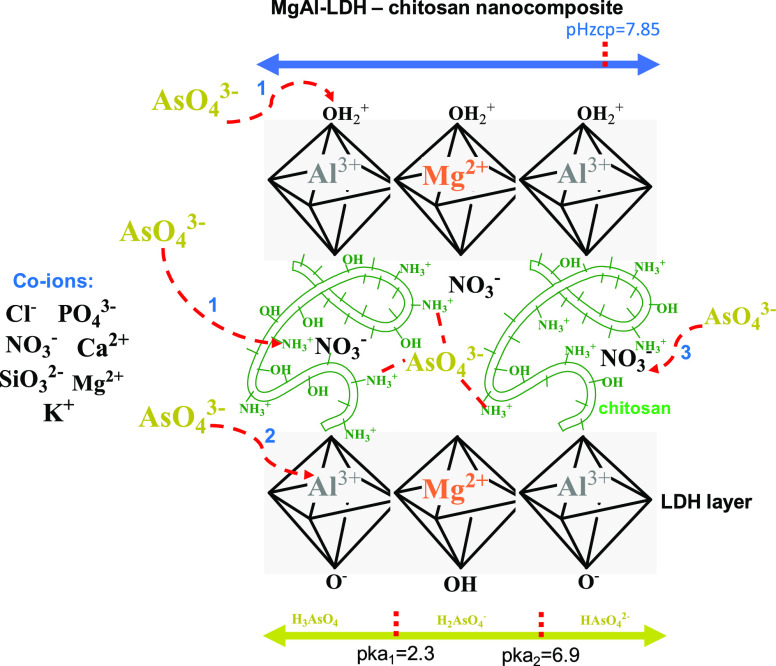
Feasible mechanisms of As(V) using Mg–Al LDH-chitosan nanocomposite.

The acid–base behavior of the adsorbent agrees with the determined zero charge point pHzcp = 7.85 for the nanocomposite (Figure 8b). At pH < pHzcp values, the surface charge density exhibited by the adsorbent is positive, while at pH > pHzcp, the surface becomes neutral or has an anionic character.^8^ Therefore, at this low pH domain, the electrostatic repulsions could prevent the efficient adsorption of As(V) (Figure 9). The 83% removal achieved at this pH shows that this new adsorbent has an electrostatic mechanism (Figure 3b) and others that will be explained below.

The pH range of 3.5–7 was the optimal performance zone for this new adsorbent material. This can be explained considering that the predominant species of As is the semi-deprotonated one, being a strategic zone between the p*K*_a_ values of arsenic.^3,61,62^ The width of the broad pH range is a synergistic effect between the acidic and base character of the layered double hyroxide (LDH) layers and the incorporated chitosan chains. These chains generate a buffering effect that favors this optimal working window of the nanocomposite.

As anions can easily undergo various removal mechanisms with MgAl-LDH-chitosan nanocomposite:

(1) Arsenate ions can be adsorbed on the surface of LDH-chitosan through electrostatic interaction.
(2) Nitrate ions in the inter layer of the adsorbent get exchanged with arsenate ions in the solution. In other words, adsorption of As(V) is through an ion-exchange mechanism.

At pH values > p*K*_a_ (6.9), the As(V) species with the highest negative charge predominates, which could favor the interaction of these anions with the positive charge density of the LDH layers.^3^ However, this demands a greater amount of cationic sites of the adsorbent, and considering its pH_zcp_ of 7.85, it could compromise the deprotonation of some sites of the chitosan chains and the superficial groups of the LDH layers, disfavoring the removal of As(V).

In addition, the high concentration of OH plays a competition on the adsorption sites and causes a significant decrease in the separation process.

Figure 8c,d shows the XRD and FTIR patterns before and after As(V) adsorption. Decrease in the intensity of the bands (1554, 1404, 1062, and 1017 cm^–1^) in the IR spectrum of arsenic adsorbed material, indicating the key functional groups involved in the removal of As(V), according to the mechanisms postulated for this new nanocomposite (see Figure 9). The decreased intensity of the band observed at 1400 cm^–1^ of N=O vibration^46,48,49^ is associated with an anion exchange process between As(V) and NO_3_^–^, leading to a decrease in the nitrate concentration in this interlayer region.^49,63^ The appearance of the new bands at 1426 and 1377 cm^–1^ due to the asymmetric stretching vibration of the As–O bond^60,62^ confirms the adsorption of As(V).

The shift and decrease in the region of the −OH band (3450 and 910 cm^–1^) show that the LDH surface also participates in the adsorption of As(V) through external complexation.^4,62^ Another possible interaction is through the -NH_2_ groups of the chitosan chains, which at these pH conditions have a cationic character and favor their electrostatic attraction of the As(V) anions. This is confirmed by the changes in the band at 1585 and 3352 cm^–1^ after adsorption on MgAl-LDH-chitosan.^5,47^ The XRD pattern before and after adsorption maintains the same characteristic peaks, showing that the adsorbent surface was not altered by As adsorption.^5^ However, the peaks become less intense and broader, which could be associated with incorporating As(V) anions on the LDH interlayers (see Figure 9). The presence of As(V) adsorbed on the surface of CsC@MgAl-LDH was also confirmed by EDS analysis, as shown in Figure S1c.

### 3.8 Antimicrobial Activity

Chitosan is already reported in many scientific studies to have many biological activities including an antibacterial effect against pathogenic bacteria. However, these bacteria can develop resistance against this polymer if used regulary. Finding a composite based on chitosan is really helpful in avoiding such issues; the new composite can be used in many pharmaceutical preparations for treatment of some infections and also in cosmetics.

**Figure 10:**
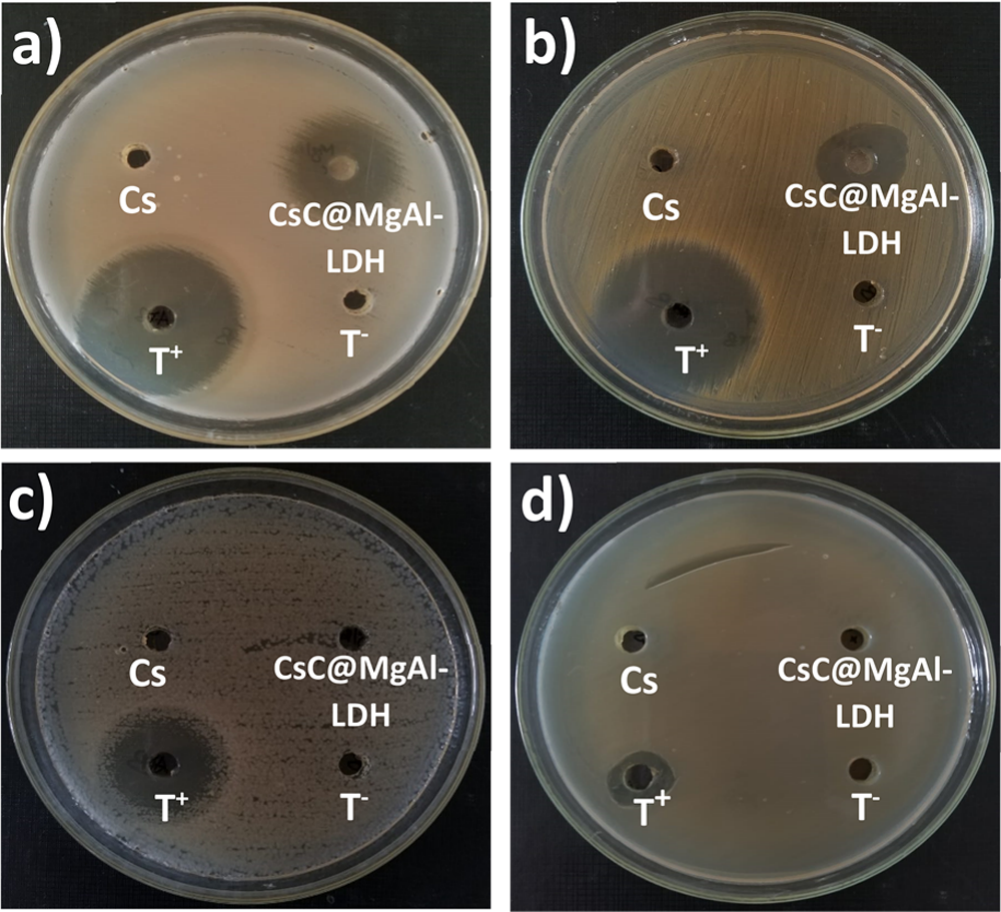
Antibacterial activity of CsC@MgAl-LDH against *Bacillus subtilus* (a), *Staphylococcus aureus* (b), *Echerichia coli* (c), and *Pseudomonas aeruginosa* (d).

Antibacterial activity of CsC@MgAl–LDH was evaluated through an agar well-diffusing assay. As shown in Table 5 and Figure 10, the antibacterial agent exhibited an effective zone of inhibition against *Staphylococcus aureus* and *Bacillus subtilus* with an inhibitory zone of 20 and 21 mm, respectively. On the other hand, there was no effect against *Pseudomonas aeruginosa* and *Escherichia coli* compared to the positive control. It is reported in the literature that the polycationic structures of chitosan are responsible for its antibacterial activity.^41−43^ Electrostatic interactions between positively charged chitosan amino groups and negatively charged elements of cell membranes cause damage in normal cell metabolism.^41,42^ Chitosan can exhibit different inhibitory efficiency effects against Gram-positive and Gram-negative bacteria, as these bacterial types^43^ have specific variations in cell surfaces.

**Table 5:** Zone of Inhibition

	Diameter of the inhibitory zone (mm)			
	*B. Subtilus*	*S. aureus*	*E. coli*	*P. aeruginosa*
CsC@MgAl–LDH	21 ± 1	20 ± 1	0	0
Chlortetracycline	32 ± 1	33 ± 1	20 ± 1	13 ± 1

Gram-positive bacteria cell wall is made up of several layers of murein on which teichoic acid is attached to the surface, contributing to the cell wall’s negative charge. Similar structures are found in Gram-negative bacteria’s outer membrane called lipopolysaccharides (LPS). Raafat and colleagues suggested that first contact probably happens between chitosan macromolecules and Gram-positive bacteria’s teichoic acids, causing damage to its membrane. However, chitosan binding to lipopolysaccharides in Gram-negative bacteria cell walls could not possibly influence cell membranes, since LPS are found in the outer membrane.^20^ This can explain the results obtained in which CsC@MgAl–LDH had no activity against the two tested Gram-negative bacteria. Then, the new composite can be used in many pharmaceutical preparations for treatment of some infections and also in cosmetics.

## 4 Conclusion

The adsorbent CsC@MgAl-LDH showed an effective adsorbent for As(V) uptake from aqueous solution attaining maximum sorption capacity up to 69.29 mg g^–1^ at 60 °C. The adsorption of As(V) was spontaneous at all the studied temperatures. The value of Δ*H*° was found to be +10.93 kJ mol^–1^, which confirms an endothermic process. The positive values of Δ*S*° indicated a more organized state of As(V) onto the activated sites of the adsorbent after its uptake. The effects of interferents using several anions showed 15–18% interference for nitrate, chloride, and phosphate anions and 10% for silicate. The adsorbent’s recyclability was efficient, maintaining at least 85% recovery after the seventh cycle of adsorption/desorption. MgAl-LDH-loaded cross-linked chitosan exhibited good performance for As(V) adsorption and as an antimicrobial agent against *Staphylococcus aureus* and *Bacillus subtilus*; this enhances the multifunctional applicability of these new hybrid compounds using biopolymers and MgAl-LDH.

## Supporting Information Available

SEM micrograph of CsC and CsC@MgAl-LDH, EDS spectrum of CsC and CsC@MgAl-LDH, Adsorption–desorption curve, and BJH pore volume (PDF)
