# Supplementary material for: Multifunctional Cross-Linked Shrimp Waste-Derived Chitosan/MgAl-LDH Composite for Removal of As(V) from Wastewater and Antibacterial Activity
Source: ACS Omega. 2023 Mar 8;8(11):10051–61. doi: 10.1021/acsomega.2c07391 (PMC10034834; doi:10.1021/acsomega.2c07391)
Supplement: Supplementary file 1 — ao2c07391_si_001.pdf [file ao2c07391_si_001.pdf]

Supporting Information

Multifunctional cross-linked Shrimp waste-derived Chitosan/MgAl-LDH composite for removal As (V) from wastewater and antibacterial activity

Rachid El Kaim Billah[1*], Zineb Azoubi[2], Eduardo Alberto López-Maldonado[3*], Hicham Majdoubi[4], Hassane Lgaz[5], Eder C. Lima[6], Anita Shekhawat[7], Youssef Tamraoui[4], Mahfoud Agunaou[1], Abdessadik Soufiane[1], Ravin Jugade[7]

[1]*Department of Chemistry, Faculty of Sciences, Laboratory of Coordination and Analytical Chemistry, University of Chouaib Doukkali, El Jadida, Morocco.*

[2]*Laboratory of Physiopathology and Molecular Genetics, Faculty of Sciences Ben M'Sick, Hassan II University of Casablanca.*

[3]*Faculty of Chemical Sciences and Engineering, Autonomous University of Baja, California, CP, 22390, Tijuana, Baja California, Mexico*

[4]*Materials Science energy and Nanoengineering Department,* Said *Mohamed VI Polytechnic University, Benguerir, Morocco.*

[5]*Department of Architectural Engineering, Hanyang University-ERICA, 1271 Sa 3-dong, Sangrok-gu, Ansan 426791, Republic of Korea*

[6]*Institute of Chemistry, Federal University of Rio Grande do Sul (UFRGS), Porto Alegre, RS, 91501-970, Brazil*

[7]*Department of Chemistry, RTM Nagpur University, Nagpur, India*

E-mail addresses of the corresponding authors: rachidelkaimbillah@gmail.com (REB), elopez92@uabc.edu.mx (EALM).

Table of contents:

Figure S1. a. SEM micrograph of CsC, b. SEM micrograph of CsC@MgAl-LDH, c. EDS spectrum of CsC, and d. EDS spectrum for CsC@MgAl-LDH

Figure S2. Adsorption-desorption curve a) and BJH pore volume b).

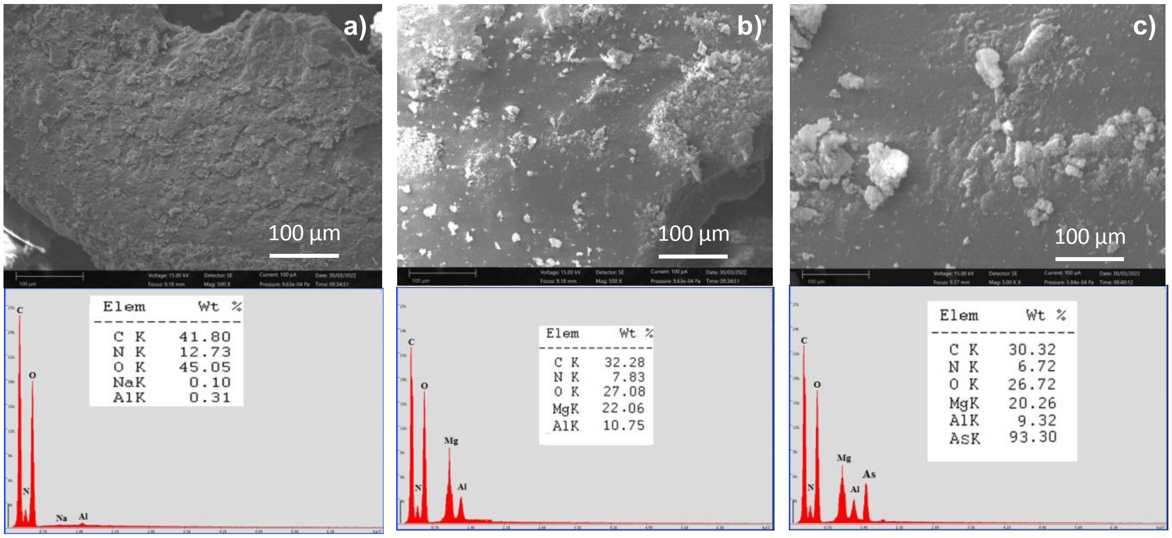

Figure S1. SEM micrograph and EDS of CsC a), CsC@MgAl-LDH b) and after As(V) adsorption CsC@MgAl-LDH c).

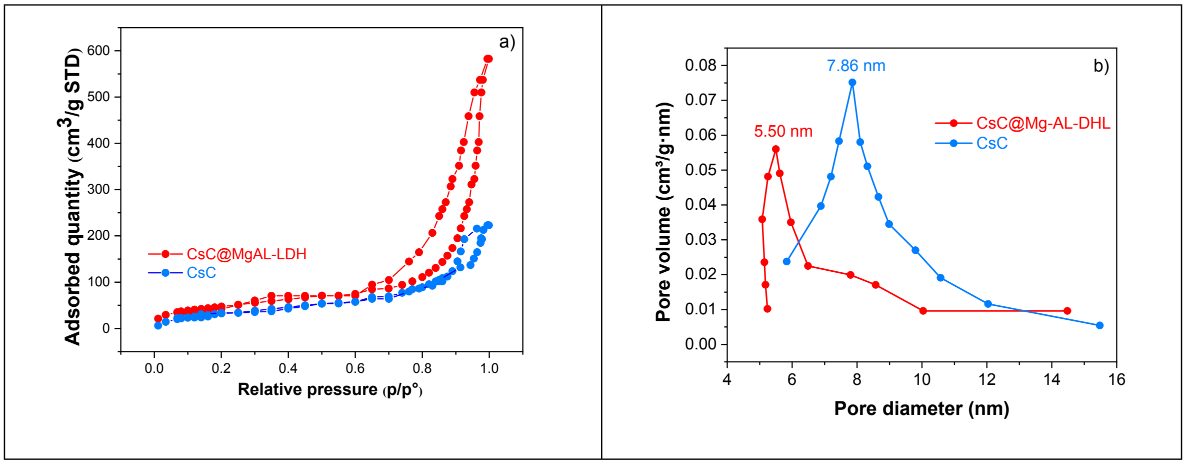

Figure S2. Adsorption-desorption curve a) and BJH pore volume b).
